# Synbiotic Supplementation Containing Whole Plant Sugar Cane Fibre and Probiotic Spores Potentiates Protective Synergistic Effects in Mouse Model of IBD

**DOI:** 10.3390/nu11040818

**Published:** 2019-04-11

**Authors:** Tanvi Shinde, Agampodi Promoda Perera, Ravichandra Vemuri, Shakuntla V. Gondalia, Avinash V. Karpe, David J. Beale, Sonia Shastri, Benjamin Southam, Rajaraman Eri, Roger Stanley

**Affiliations:** 1 Centre for Food Safety and Innovation, Tasmanian Institute of Agriculture, University of Tasmania, Launceston, TAS 7250, Australia; 2 School of Health Sciences, College of Health and Medicine, University of Tasmania, Launceston, TAS 7250, Australia; agampodi.perera@utas.edu.au (A.P.P.); ravichandra.vemuri@utas.edu.au (R.V.); sonia.shastri@utas.edu.au (S.S.); benjamin.southam@utas.edu.au (B.S.); rajaraman.eri@utas.edu.au (R.E.); 3 Centre for Human Psychopharmacology, Swinburne University of Technology, Hawthorn, VIC 3122, Australia; sgondalia@swin.edu.au; 4 Land and Water, Commonwealth Scientific and Industrial Research Organization (CSIRO), Ecosciences Precinct, Dutton Park, QLD 4102, Australia; Avinash.Karpe@csiro.au (A.V.K.); David.Beale@csiro.au (D.J.B.)

**Keywords:** synbiotic, prebiotic, probiotic, IBD, *Bacillus* spores, dietary fibre, sugar cane fibre, ulcerative colitis

## Abstract

Inflammatory bowel diseases (IBD) are a chronic inflammatory disorders with increasing global incidence. Synbiotic, which is a two-point approach carrying probiotic and prebiotic components in mitigating inflammation in IBD, is thought to be a pragmatic approach owing to the synergistic outcomes. In this study, the impacts of dietary supplementation with probiotic *Bacillus coagulans* MTCC5856 spores (*B. coagulans*) and prebiotic whole plant sugar cane fibre (PSCF) was assessed using a murine model of IBD. Eight-week-old C57BL/6 mice were fed a normal chow diet supplemented with either *B. coagulans*, PSCF or its synbiotic combination. After seven days of supplementation, colitis was induced with dextran sulfate sodium (DSS) in drinking water for seven days during the continuation of the supplemented diets. Synbiotic supplementation ameliorated disease activity index and histological score (−72%, 7.38, respectively), more effectively than either *B. coagulans* (−47%, 10.1) and PSCF (−53%, 13.0) alone. Synbiotic supplementation also significantly (*p* < 0.0001) prevented the expression of tight junction proteins and modulated the altered serum IL-1β (−40%), IL-10 (+26%), and C-reactive protein (CRP) (−39%) levels. Synbiotic supplementations also raised the short-chain fatty acids (SCFA) profile more extensively compared to the unsupplemented DSS-control. The synbiotic health outcome effect of the probiotic and prebiotic combinations may be associated with a synergistic direct immune-regulating efficacy of the components, their ability to protect epithelial integrity, stimulation of probiotic spores by the prebiotic fibre, and/or with stimulation of greater levels of fermentation of fibres releasing SCFAs that mediate the reduction in colonic inflammation. Our model findings suggest synbiotic supplementation should be tested in clinical trials.

## 1. Introduction

Inflammatory bowel disease (IBD) is a chronic relapsing inflammatory condition of the gastrointestinal tract that comprises two partially overlapping but distinct clinical entities: Crohn’s disease (CD) that involves the entire gastrointestinal tract and ulcerative colitis (UC) that is limited to colon and rectum [1]. The incidence of CD and UC has become a global disease with accelerating incidence in countries adopting a Westernised diet, highlighting the urgent need for research into prevention and management of this complex and costly pathology [2]. Although the aetiology and pathogenesis of IBD still remains unclear, emerging evidence supports the involvement of a recurrent tripartite pathophysiological circuit encompassing gut dysbiosis, altered epithelial integrity and defective immune responses [1]. Therefore, preventive and therapeutic approaches that impede or break this inflammatory circuit by resolving one or more of the pathophysiological circuit components are highly sought. 

Dietary interventions are increasingly perceived as both preventive and corrective strategies to normalise the dysfunctional microbiome, altered immune and barrier integrity functions to normality in IBD [3,4,5]. In this regard, probiotic and prebiotic dietary fibres (DF) are thought to be useful in mitigating the inflammatory circuit thereby resolving or preventing the severity of IBD. Both bioactive ingredients can improve inflammatory parameters in the gut by modifying microbiota composition and metabolites, regulating secretion of immunomodulatory molecules and protecting the colonic epithelial barrier [1,6,7,8]. Synbiotics, being a combination of probiotic and prebiotic ingredients that positively interact, potentially offer prophylactic and therapeutic effects that could function synergistically to confer health benefits to the host.

DFs have shown particular promise in attenuating colonic inflammation in humans [9]. Meanwhile, the underlying mechanisms are likely to be multifactorial: they include dilution of toxins via stool bulking and the production of metabolites, particularly short-chain fatty acids (SCFA), as a result of microbial fermentation is frequently cited as a major potential contributor to the protective effect [10]. DFs consist of edible plant parts that resist digestion and absorption in the small intestine and undergo complete or partial fermentation in the colon. It is an extremely complex group of substances, including non-starch polysaccharides, resistant starch, cellulose and hemicellulose, oligosaccharides, pectins, gums, lignin, and waxes [11]. Much work on DFs, however, has examined various purified ingredients that represent limited chemical complexity, contrasting to those that naturally occur in fruits and vegetables [12]. Nevertheless, the biochemical complexity of DFs is recently being more appreciated to be a vital factor influencing the gut microbial complexity [12,13,14]. This highlights the prudence of application of prebiotic fibres that are representative of whole plant vegetables and fruits, prepared to retain fibre biochemical complexity. In this context, a process to produce sugar cane fibre by wet diffusion to remove most of the sucrose from cut cane, which is then dried and ground into a flour, has been reported to preserve the cell wall components and retain other intrinsic nutritional biologically active components [15,16]. Such fibre, in addition to retaining other micronutrients and polyphenols, also contains both soluble and insoluble benefits as well as rapid- and poor-fermentable fibres and at ratios that more accurately represent natural whole plant foods. In a recent study, such sugar cane fibre has been shown to impart positive effects on human gut microbiota in vitro [17]. The high content of total dietary fibre (87%) was accounted for with respect to its positive effect in this study. The relative similarity of this sugar cane fibre product to that in other whole plant foods indicates its potential as a convenient supplementary source of dietary fibre that could alter microbial ecology and have a positive influence for IBD attenuation. 

*Bacillus coagulans* is a GRAS (generally recognised as safe) affirmed probiotic that can ferment a variety of plant substrates rich in insoluble cell wall components [18,19] more efficiently than most members of gut microbiota [20,21]. It is also known to be capable of modulating the innate immune system by binding and interacting with the gastrointestinal tract epithelium [22,23]. This makes it a suitable probiotic for its synbiotic combination with prebiotic whole plant sugar cane fibre (PSCF) rich in insoluble cell wall fractions. Probiotic *Bacillus coagulans* MTCC (Microbial Type Culture Collection) 5856 spores in addition to exhibiting excellent immunomodulatory effects in vitro, have shown significant survival during simulated gastric transit with substantial adhesion capacity to human colonic epithelial cells [22]. Based on the aforementioned findings, we hypothesised that preconditioning with probiotic *B. coagulans* MTCC 5856 spores, prebiotic PSCF and its synbiotic combination might repress the onset and/or severity of DSS-induced colitis in mice. This study aimed to evaluate the efficacy of probiotic *B. coagulans* spores and PSCF, both alone and in combination as synbiotic to ameliorate the onset of experimental colitis in mice and further examine its underlying mechanisms. 

## 2. Materials and Methods

### 2.1. Probiotic Bacteria and Prebiotic Dietary Fibre

LactoSpore^®^ (Sabinsa Corporation, East Windsor, NJ, USA) containing the probiotic strain *Bacillus coagulans* MTCC 5856 (6 × 10^9^ spores/gm) was produced by Sami Labs Limited (Bangalore, India) and supplied by Sabinsa Corporation (Australia). Kfibre™, prebiotic whole plant sugar cane fibre (PSCF) was supplied by KFSU Pty Ltd., Queensland, Australia. 

### 2.2. Animals

Fifty C57BL/6J (seven week old) mice of both sexes of average weight 19g were obtained from the University of Tasmania animal breeding facility and housed in a temperature-controlled environment with a 12-h day/night light cycle. Individual body weights were assessed daily including over an initial acclimation period of seven days. All mice had ad libitum access to radiation-sterilised rodent feed pellets (Barastoc Rat and Mouse, Ridley AgProducts, Melbourne, Victoria, Australia) and autoclaved tap water for drinking during experiments. All animal experiments were approved by the Animal Ethics Committee of the University of Tasmania (ethics approval number: A0015840) and conducted in accordance with the Australian Code of Practice for Care and Use of Animals for Scientific Purposes (8th Edition, 2013). All efforts were made to minimize animals’ suffering and to reduce the number of animals used. 

### 2.3. Study Design and Treatments

Following one week of acclimation, mice at eight weeks of age were randomly allocated into the following five groups (*n* = 10 per group): (1) Healthy control (HC), (2) DSS-control, (3) probiotic *B. coagulans* MTCC 5856 spores (*B. coagulans*), (4) whole plant prebiotic sugar cane fibre (PSCF) supplement, and (5) synbiotic combined supplement. Mice in HC and DSS-control groups received 4 g chow mash (standard chow pellet blended with water). The *B. coagulans* group received 4 g chow mash supplemented with probiotic *B. coagulans* MTCC 5856 spores (2 × 10^9^ CFU/day/mouse). The PSCF group received 4g chow mash supplemented with Kfibre™ (200 mg/day/mouse). Synbiotic treatment group mice received 4 g chow, each supplemented with *B. coagulans* MTCC 5856 spores (2 × 10^9^ CFU/ day/mouse) and Kfibre (200 mg/day/mouse). The chow mash was prepared fresh each day. The mice were single-caged throughout the experiment to measure the defined daily intake of respective treatments from prepared chow mash. The mice were fed these treatments for 14 days. Colitis was induced during the last seven days of the experimental period as previously described [24], by administering 2% dextran sulfate sodium (DSS; MP Biomedicals, colitis grade average molecular weight: 36,000–50,000) in the drinking water of all groups except for the non-colitic DSS-control mice which received normal drinking water. Mice were sacrificed on day 15 by CO_2_ asphyxiation. 

### 2.4. Clinical Scoring and Histological Analysis

A Disease Activity Index (DAI) was determined daily in all mice by scoring for body weight, hemocult reactivity or presence of gross blood and stool consistency during the week of DSS induction, as detailed in [25]. Stool was collected from individual mice and tested for the presence of blood using Hemoccult II slides (Beckman Coulter Inc., Brea, CA, USA). Briefly, the following parameters were used for calculation: (a) body weight loss (score 0 = 0%, score 1 = 1–5%, score 2 = 6–10%, score 3 = 11–15%); (b) stool consistency (score 0 = normal, score 1 = soft but still formed, score 2 = very soft/loose stool, score 3 = diarrhoea/watery stool); and (c) blood in stool (score 0 = negative hemoccult, score 1 = positive hemoccult, score 2 = Blood traces in stool visible, score 3 = rectal bleeding). DAI was determined by combining the scores from these three categories. Body weights were measured for each animal throughout the experiments and expressed as percent weight loss to the weight immediately before DSS treatment. Fecal samples were collected and stored at −80 °C on day 14 for metabolite analysis.

After sacrificing the mice, the colons were removed from the caecum to the anus following the method of Perera et al. [26]. The length of the colons from the ileocaecal junction to the rectum were recorded. The colon was subsequently opened along its longitudinal axis and the luminal (mucosal) contents were removed using sterilised 200 μL pipette tips prior to weighing the organ. The length and weight of colon and spleen were documented. Spleen weight, colon length, and colon weight/body weight ratio were calculated as macroscopic markers of inflammation. The mucosal and cecal contents were collected for metabolite profiling and stored at −80 °C. The colon was bisected longitudinally, and one half was prepared using the Swiss roll technique [27] whereas the remaining colonic tissue was dissected out, segregated into proximal colon (PC) and distal colon (DC) and snap-frozen for molecular analyses. Swiss rolls underwent 24 h fixation in 10% (*v/v*) neutral-buffered formalin. Swiss rolls were subsequently transferred to 70% ethanol prior to progressive dehydration, clearing and infiltration with HistoPrep paraffin wax (Fisher Scientific, Philadelphia, PA, USA). Swiss rolls were then embedded in wax and 5 μm sections were cut using a rotary microtome. Sections were stained with haematoxylin and eosin (H and E; HD Scientific, Sydney, Australia). Slides stained with H and E (*n* = 8 per group) graded blindly for the severity of tissue damage at distal and proximal regions as described previously [28,29]. Briefly, frequency of distribution of inflammation graded 0-3, crypt architectural distortion and ulceration graded 0–5, tissue damage graded 0-3, inflammatory infiltrate graded 0–3, goblet cell loss graded 0–3, mucosal thickening (oedema) were graded 0–3. All images were captured on a Leica DM500 microscope using a Leica ICC50 W camera (Leica Microsys-tems, Wetzlar, Germany). 

### 2.5. Alcian Blue Staining

DSS-induced alterations in goblet cells and subsequent depletion in synthesis and secretion of mucin glycoprotein (MUC2) was analysed by Alcian blue staining (ab150662 Alcian Blue, pH 2.5 (Mucin Stain), Abcam, Australia) following the manufacturer’s instructions. Briefly, paraffin-embedded colon sections (*n* = 4/group) were stained with Alcian blue, staining the acidic sulphated mucin blue and the counterstained with Safranin O, staining the nuclei red following the method previously described [30]. Computer-assisted image analysis was performed with a Leica DM500 microscope (Leica Microsystems, Wet-zlar, Germany) and Leica ICC50 W camera (Leica Microsys-tems, Wetzlar, Germany). The staining intensity (IOD) was assessed using Image Pro Plus 7.0 (Media Cybernetics, Inc., Rockville, MD, USA) and used for comparison among groups [31]. 

### 2.6. Immunohistochemical Detection of Tight Junction Proteins

Immunohistochemical detection of epithelial tight junction (TJ) proteins was performed using a Rabbit specific HRP/DAB (ABC) Detection IHC kit (ab64261, Abcam, Australia) following the manufacturer’s instruction and as previously described [32]. Following removal of paraffin and rehydration, the tissue sections were exposed to heat-induced epitope retrieval (4 min at 121 °C) in a sodium citrate buffer, pH 6 in a Decloaking chamber (Biocare Medical, Pachico, CA, USA). After washing the slides in 1× phosphate buffered saline (PBS) 2 mins/wash, endogenous peroxidase activity was blocked by incubating the slides with hydrogen peroxide block for 10 min. Next, the slides were washed with PBS (2 × 2 min) washes and protein block was then applied for 30 min at room temperature to block non-specific background staining. Following PBS (1 × 2 min) wash, colon sections were then incubated with primary antibodies: anti-ZO-1 (NBP1-85046, Novus Biologicals, Australia, 1:400); anti-occludin (NBP1-87402, Novus, 1:600); anti-claudin-1 (NBP1-77036, Novus, 1 μg/mL) overnight at 4 °C. Sections were then washed with PBS (4 × 2 min) and biotinylated goat anti-rabbit IgG was applied and incubated for 10 min at room temperature. At the end of incubation, the slides were washed in PBS (4 × 2 min) and streptavidin peroxidase was applied to the sections and further incubated for 10 at room temperature. The slides were then thoroughly rinsed with PBS (4 × 2 min) before sections were covered with 3,3′-diaminobenzidine (DAB) chromogen and substrate solution for 10 min. Tissue sections were subsequently counterstained with hematoxylin, dehydrated, and mounted with DPX media (Sigma-Aldrich, Sydney, Australia).

Computer-assisted image analysis was performed with a Leica DM500 microscope (Leica Microsystems, Wet-zlar, Germany), Leica ICC50 W camera (Leica Microsys-tems, Wetzlar, Germany), and Image Pro Plus 7.0 (Media Cybernetics, Inc., Rockville, MD, USA) software. The expression of tight junction proteins (TJPs): ZO-1, occludin and claudin-1 was blindly assessed by choosing random five fields on each slide (*n* = 4/group). Barrier TJ protein expressions and staining intensity in colonic epithelium was expressed as the percentage expression of a respective TJ protein. 

### 2.7. Myeloperoxidase Activity

The extent of the inflammatory cell invasion in the colon was examined by the assessment of myeloperoxidase (MPO) activity [24]. Weighed and snap frozen PC and DC specimens (*n* = 3) were analysed for MPO activity using a Myeloperoxidase Activity Assay kit (ab105136, colorimetric, Abcam^®^, Cambridge, UK). Briefly, frozen tissue after washing in cold PBS, was resuspended in MPO assay buffer, before homogenisation with 10–15 passes using an Omni TH tissue homogeniser (Omni International, US) with 10–15 passes. The homogenate was then centrifuged at 13,000× *g* (10 min) and the supernatant assayed for MPO activity as per the manufacturer’s instructions. The values are expressed as MPO activity units/g tissue. 

### 2.8. Tissue Explant Culture and Cytokine Measurements

PC and DC colon tissues of mice from each group were cut, weighed and washed with cold PBS before transferring to a 12 well plate containing 0.5 mL/well of RPMI1640 culture medium (In Vitro Technologies Pty Ltd, Melbourne, Australia) supplemented with 10% *v/v* fetal calf serum (Gibco, Life Technologies Pty Ltd, Melbourne, Australia), penicillin (100 mU/L), and streptomycin (100 mg/L) (Sigma-Aldrich Pty Ltd, Sydney, Australia) as described previously [26]. After 24 h of incubation, supernatant was collected from each well, centrifuged and stored at −80 °C until further analysis. Serum was collected from blood drawn by cardiac puncture at the end of the study for cytokine analysis.

The cytokine levels in colon tissue (*n* = 3) and serum (*n* = 3) were determined by immunoassay using a Bio-Plex Pro Mouse cytokine 23-plex kit (Bio-Rad #M60009RDPD, Bio-Rad Laboratories, Gladesville, NSW, Australia) following the manufacturer’s instructions and concentrations analysed using a Bio-Plex 200 instrument (Bio-Rad) and Bioplex Manager software, version 6 (Bio-Rad Laboratories) respectively. For tissues, the cytokine levels were normalized by dividing the cytokine results (pg/mL) by the measured biopsy weight (g). The most significantly altered cytokines are presented as pg/g of tissue.

### 2.9. iNOS Activity

The expression of inducible isoform of nitric oxide synthase (iNOS) in colonic epithelial cells in response to pro-inflammatory stimuli [33] was determined in PC and DC specimens using a Nitic Oxide Synthase Activity Assay kit (ab211084, Fluorometric, Abcam^®^, Cambridge, UK). Snap frozen proximal and distal colonic tissues (*n* = 3) were washed in in cold PBS and resuspended in 200 μL NOS assay buffer then homogenised by 10–15 passes of an Omni TH tissue homogeniser (Omni International, Tulsa, OK, USA). The homogenate was then centrifuged at 10,000× *g* (10 min, 4 °C) and the supernatant then underwent iNOS activity assay activity assay as per the manufacturer’s instructions. The amount of protein in the lysate was determined using *DC*™ Protein Assay (Bio-Rad Laboratories, Australia). The results are expressed as iNOS activity mU/mg. 

### 2.10. Serum C-Reactive Protein Analysis

The levels of C-reactive protein (CRP) in serum from respective groups (*n* = 3 samples/group) were analysed using Mouse C-Reactive Protein/CRP Quantikine Elisa kit (MCRP00, R and D Systems, Australia) following the manufacturer’s instructions. The results are expressed as μg/mL.

### 2.11. Volatile SCFA Analysis

Cecal, mucosal and fecal samples (*n* = 5 per group) were prepared and derivatized following the protocol developed by Furuhashi et al. [34] with some modifications. Briefly, cecal, mucosal and fecal samples of 100–150 mg fresh weight (stored at −80 °C) were weighed to ± 0.1 mg accuracy. These samples were added to a sterile 1.5 mL bead-beating tube (NAVY Rino Lysis tubes, Next Advance, Troy, NY, USA). A 1.0 mL aliquot isobutanol (10% MilliQ water), (LC-MS grade, Merck, Castle Hill, NSW, Australia) was added to each sample, followed by two 30 s, 4000 rpm homogenization pulses sandwiched between a 20-s pause interval (Precellys Evolution Homogenizer, Bertin Instruments, Montigny-le-Bretonneux, France). The samples were subsequently centrifuged at 16,000× *g* for 6 min.

The supernatant (675 µL) was transferred to a clean round bottomed 2 mL centrifuge tube (Eppendorf South Pacific Pty. Ltd., Macquarie Park, NSW, Australia) and NaOH (20 mM, 125 µL, Merck Pty Ltd., Castle Hill, NSW, Australia) and chloroform (400 µL, LC-MS grade, Merck Pty Ltd.,) were added. The samples were briefly vortexed and centrifuged at 16,000·*g* for 3 min. The aqueous phase (upper layer, 400 µL) was transferred to a new clean round bottomed 2 mL centrifuge tube (Eppendorf South Pacific Pty. Ltd., Macquarie Park, NSW, Australia) containing a boiling chip (Sigma Aldrich, Castle Hill, NSW, Australia). Pyridine (100µL), isobutanol (80 µL) (both LC-MS grade, Sigma Aldrich, Castle Hill, NSW, Australia), and MilliQ (Millipore Corporation) water (70 µL) were added and the samples were subjected to gentle hand vortexing (swirling action) followed by the addition of 50 µL isobutyl chloroformate (98% purity, Sigma Aldrich, Castle Hill, NSW, Australia). The tube was kept opened to release any generated gases and was allowed to stand for about one minute. Hexane (150 µL, LC-MS grade, Sigma Aldrich, Castle Hill, NSW, Australia) was then added to each tube, which was then capped and vortexed prior to centrifugation at 15,700× *g* for 4 min. The upper phase (100 µL) was subsequently transferred to clean gas chromatography (GC) autosampler vial fitted with silanized low volume glass inserts; Malathion (1 µL, equivalent to 2.5 µg dry weight) was added as an internal standard.

The GC-MS analysis was performed on an Agilent 6890B GC oven coupled to a 5977B mass spectrometer (MS) detector (Agilent Technologies, Mulgrave, VIC, Australia) fitted with an MPS autosampler (Gerstel GmbH & Co.KG, Mülheim an der Ruhr, Germany). The GC oven was fitted with two 15 m HP-5MS columns (0.25 mm ID and 0.25 µm film thickness; 19091S-431 UI (Ultra Inert), Agilent Technologies, VIC, Australia) coupled to each other through a purged ultimate union (PUU) for the use of post-run backflushing. The sample (1.0 µL) was introduced via a multimode inlet (MMI) operated in split mode (1:20). The column was maintained at 40 °C for 5 min, followed by an increase to 250 °C at a rate of 10 °C/min. This was followed by a second increment to 310 °C at a rate of 60 °C/min. The column was held at 310 °C for 1 min. The mass spectrometer was kept in extractor ion mode (EI mode) at 70 eV. The GC-MS ion source temperature and transfer line were kept at 250 °C and 280 °C, respectively. Detector voltage was kept at 1054 V. The MS detector was turned off for the first 3 min and, at 4.0–4.8 min and 12.5–13.2-min time windows until the excess derivatization reagent (chloroformate/hexane solvents) were eluted from the column. This ensured that the source filament was not saturated and damaged. The scan range was kept in the range of *m/z* 35–350 (35–350 Daltons). Data acquisition and spectral analysis were performed as described in a previous study [35] and qualitative identification of metabolites was performed according to the Metabolomics Standard Initiative (MSI) chemical analysis workgroup [36] using standard GC-MS reference metabolite libraries (NIST 17, and an in-house CF-based metabolomics library developed after Smart et al. [37] with the use of Kovats retention indices based on a reference n-alkane standard (C8-C40 Alkanes Calibration Standard, Sigma-Aldrich, Castle Hill, NSW, Australia).

### 2.12. Metabolic Phenotyping Analysis

Untargeted metabolomic profiling of cecal, mucosal and fecal samples (*n* = 5 per group) were performed using GC-MS analysis as described previously [35]. 

### 2.13. Statistical Analysis

The samples in the study were randomly chosen for all the analysis to avoid bias. All data are presented as means ± standard error of the mean (SEM). The statistical analysis was performed with the use of GraphPad Prism Software (Version 7.0, San Diego, CA, USA) The data were evaluated using One-way analysis of variance (ANOVA) followed by Tukey’s post-hoc test to determine statistical differences between the groups against the DSS-control samples. For the analysis of DAI and body weight changes during the experimental period, two-way ANOVA followed by Tukey’s post-hoc test was used, setting treatment and the time as the variables. A *p*-value of < 0.05 was considered significant. 

## 3. Results 

### 3.1. Effects of B. Coagulans, PSCF and Synbiotic Supplementation on DAI and Macroscopic Inflammatory Markers in DSS-Induced Mice

**Figure 1:**
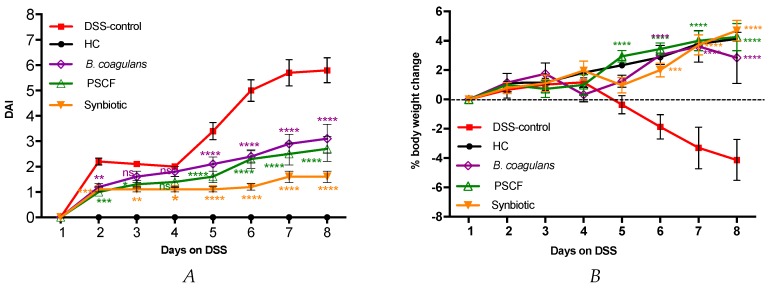

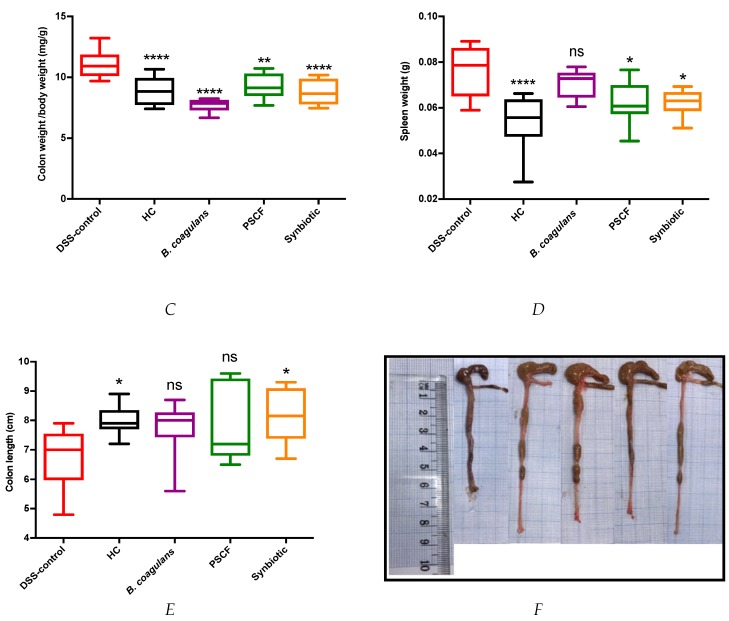
Effect of *B. coagulans* spores, PSCF and synbiotic in DSS-induced colitis model. (**A**) Disease Activity Index (DAI), (**B**) % body weight change. Statistical significance among groups evaluated by two-way repeated-measures analysis of variance (ANOVA) followed by Tukey’s test. *p < 0.05, **p < 0.01, ***p < 0.001, ****p < 0.0001 vs. DSS-control group and data expressed as mean ± SEM (*n* =10 per group). Colon weight/body weight ratio (**C**), Spleen weight (**D**), Colon length (**E**) and Macroscopic appearance of colon (**F**). Data expressed as mean ± SEM (*n* =10 per group), evaluated by one-way ANOVA followed by Tukey’s Test. ns-non-significant, HC-Healthy control, PSCF-Prebiotic sugar cane fibre.

In comparison with the HC group, the administration of probiotic, prebiotic and synbiotic treatments in the respective groups did not show any sign of toxicity, which was evaluated by body weight increase, food intake and general appearance of the animals. DAI (cumulative score for body weight change, stool consistency and blood in feces) was evaluated to determine the efficacy of the treatments in reducing the severity of disease symptoms in DSS-induced colitis (Figure 1A). Compared with the DSS-control group that showed severe colitis symptoms, pre-conditioning with *B. coagulans*, PSCF and synbiotic combination significantly reduced the DAI levels as early as day2 until the end of experiment. At the end of the experiment, DAI of DSS-control group was significantly high (5.8 ± 0.5) (*p* ≤ 0.0001) compared with that of *B. coagulans* (3.1 ± 0.6, 47% reduction), PSCF (2.7 ± 0.5, 53% reduction), and synbiotic (1.6 ± 0.2, 72% reduction) groups. DSS induction in DSS-control mice resulted in significant body weight loss until the end of experiment (−4.13 ± 1.4%). In contrast, mice maintained healthy body weight gain with *Bacillus* (2.84 ± 1.7%), PSCF (4.25 ± 1.0%), and synbiotic (4.7 ± 0.7%) treatments. Interestingly, PSCF was the more effective in reducing DAI starting from day 2 of DSS and in remediating the body weight loss as early as day 5 owing mainly to the impact of improvement in stool consistency on the DAI rating.

The macroscopic evaluation of colonic segments determined the beneficial effects of all three treatments used in our study as evidenced by substantial reduction in colon weight/body weight ratio (*B. coagulans*, 7.68 ± 0.2; PSCF, 9.24 ± 0.3 and synbiotic, 8.74 ± 0.3 mg/g) compared with DSS-control group (11.12 ± 0.3 mg/gm) (Figure 1C). Intestinal inflammation is associated with spleen enlargement [24] and, as expected, relative spleen weight of untreated DSS-control mice was significantly higher (0.076 ± 0.004 g) than that of HC mice (0.054 ± 0.003 g). PSCF (0.062 ± 0.003 g) and synbiotic (0.063 ± 0.002 g) were equally significantly effective in reducing spleen weight while *B. coagulans* did not affect the relative spleen weight (Figure 1D). In contrast to shortening of colon length (Figure 1E,F) in DSS-control group (6.75 ± 0.3 cm), synbiotic treatment proved effective in reducing this outcome by maintaining the colon length (8.12 ± 0.2 cm), significantly equal (*p* = 0.99) to that of HC group (8.01 ± 0.2 cm). These markers are considered to be directly correlated to the severity of colonic damage in this experimental model of colitis [24]. 

### 3.2. Effects of B. Coagulans, PSCF and Synbiotic Supplementation on Histological Alterations in DSS-Induced Mice

**Figure 2:**
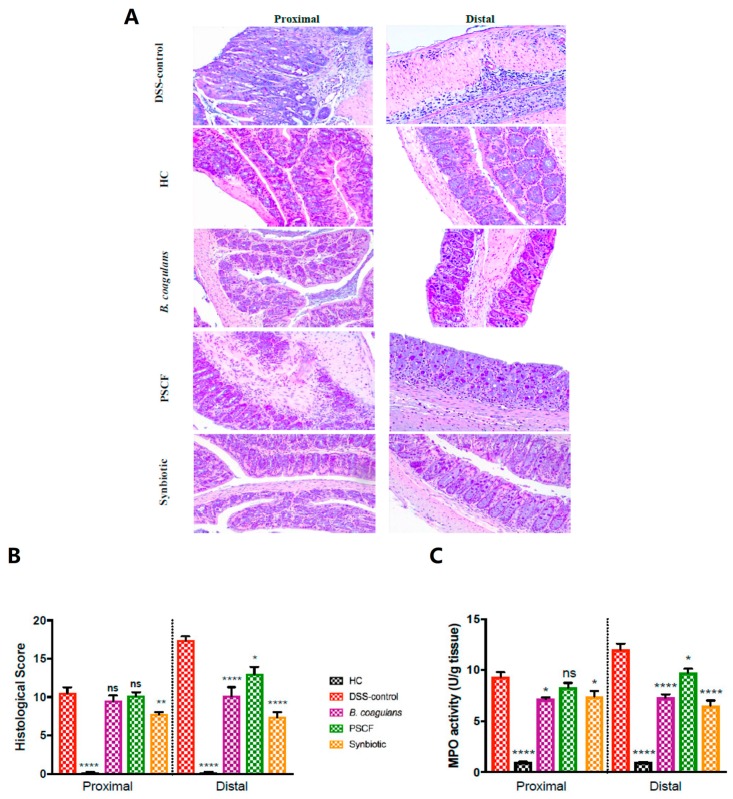
Effect of *B. coagulans* spores, PSCF and synbiotic treatments on DSS-induced colon injury and inflammation. (**A**) Histological images of proximal and distal colonic tissues stained with hematoxylin and eosin at 20× for each experimental group. (**B**) Histological score calculated after microscopic analyses of proximal and distal sections of the colon. (**C**) Myeloperoxidase (MPO) activity in colonic tissues was determined by colorimetric assay. Results expressed as mean ± SEM (*n* = 8 per group), evaluated by one-way ANOVA followed by Tukey’s test (*p < 0.05, **p < 0.01, ****p < 0.0001).

Histological (H and E staining) examination of proximal colon (PC) and distal colon (DC) sections of DSS-induced mice showed altered erosion or destruction of epithelium, crypt distortion, depletion of goblet cells, submucosal oedema, and inflammatory cellular infiltration in the colon, mostly affecting the DC (Figure 2A). HC mice showed no signs of histological colon damage with score 0, while DSS induction in DSS-control mice resulted in a cumulative damage score of 10.5 ± 0.8 for the PC and 17.4 ± 0.5 for the DC (Figure 2B). Supplementation of DSS-induced mice with *B. coagulans*, PSCF and synbiotic induced protection and repair of the colonic mucosa. *B. coagulans* and synbiotic in particular were more effective in retention of colonic structure, protection of crypts and goblet cells and rescued infiltration of inflammatory cells, which resulted in a significant overall reduction of cumulative histological score of DC (10.1 ± 1.2 and 7.38 ± 0.7 respectively). Relatively, PSCF also provided partial significant protection with histological score of 13.0 ± 1.0. In contrast, PCSF had no effect in PC (10.1 ± 0.5) with only *B. coagulans* and synbiotic treatments being significantly successful in reducing damage to the PC with histological scores of 9.5 ± 0.7 and 7.8 ± 0.3 respectively. Unlike DSS-control group, there was reduced polymorphic inflammatory infiltrate in the lamina propria and submucosa in probiotic, prebiotic and synbiotic supplemented group. This observation corroborates with the significantly reduced MPO activity in the colon (Figure 2C) of all three treatments compared with the DSS-control group. The attenuation of colonic inflammation in pre-conditioned mice (*B. coagulans*, PSCF and synbiotic) is probably due to the anti-inflammatory properties of the functional dietary ingredients tested in this study. 

### 3.3. Effects of B. Coagulans, PSCF and Synbiotic Supplementation on Goblet Cells and Colonic Tight Junction Barrier

**Figure 3:**
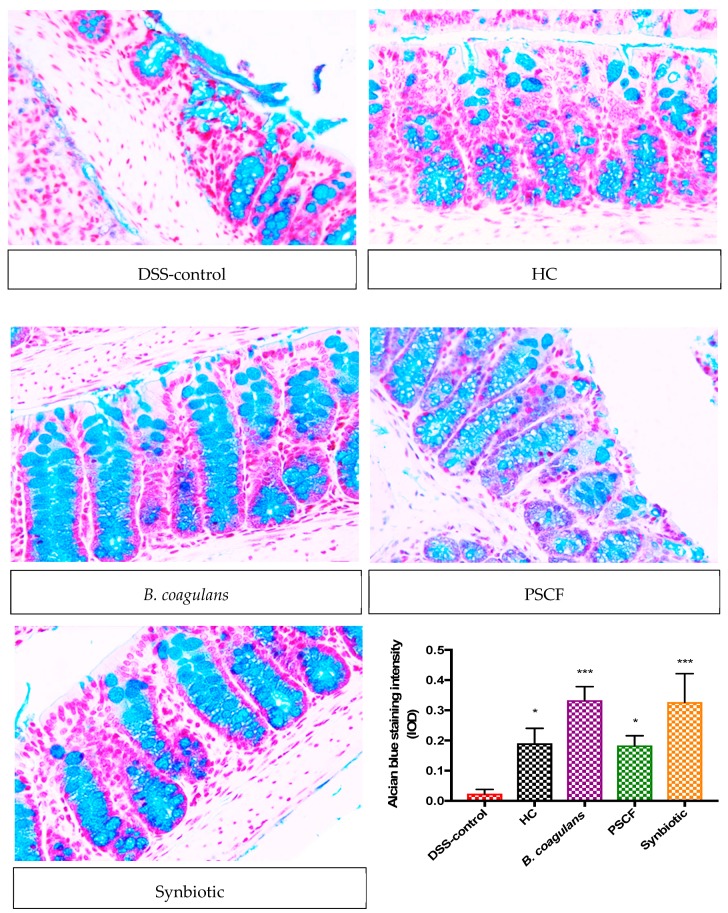
Effect of *B. coagulans* spores, PSCF and synbiotic on goblet cells. The paraffin embedded sections were stained with Alcian Blue to detect changes in goblet cells and in production of mucus in distal colonic tissue in each experimental group (40×) and staining intensity (IOD) of respective group is illustrated in the graph. Results expressed as mean ± SEM (*n* = 4 per group), evaluated by one-way ANOVA followed by Tukey’s test (*p < 0.05, ***p < 0.001).

Histological examination of distal colon from DSS mice showed a depletion of goblet cells when compared to HC and pre-conditioned mice. This suggested beneficial effects of probiotic and prebiotic ingredients on the intestinal epithelium potentially through stimulating mucus secretion by goblet cells. Specific staining with Alcian blue was therefore carried out to assess the mucus production following the administration of probiotic, prebiotic and synbiotic treatments. As depicted in Figure 3, in comparison with DSS-control group, there was a higher level of mucus staining with Alcian blue in supplemented mice samples. This implied that there has been an induction of higher levels of mucus secretion in the DSS-challenged mice that received *B. coagulans* spores, symbiotic, and PSCF supplementaions. Unlike the DSS-controlled samples where, goblet cells were almost entirely destroyed, the mice supplemented with synbiotic and *B. coagulans* showed protection of the goblet cells. PSCF also partially protected the goblet cells with mucus staining compared with DSS-control. 

**Figure 4:**
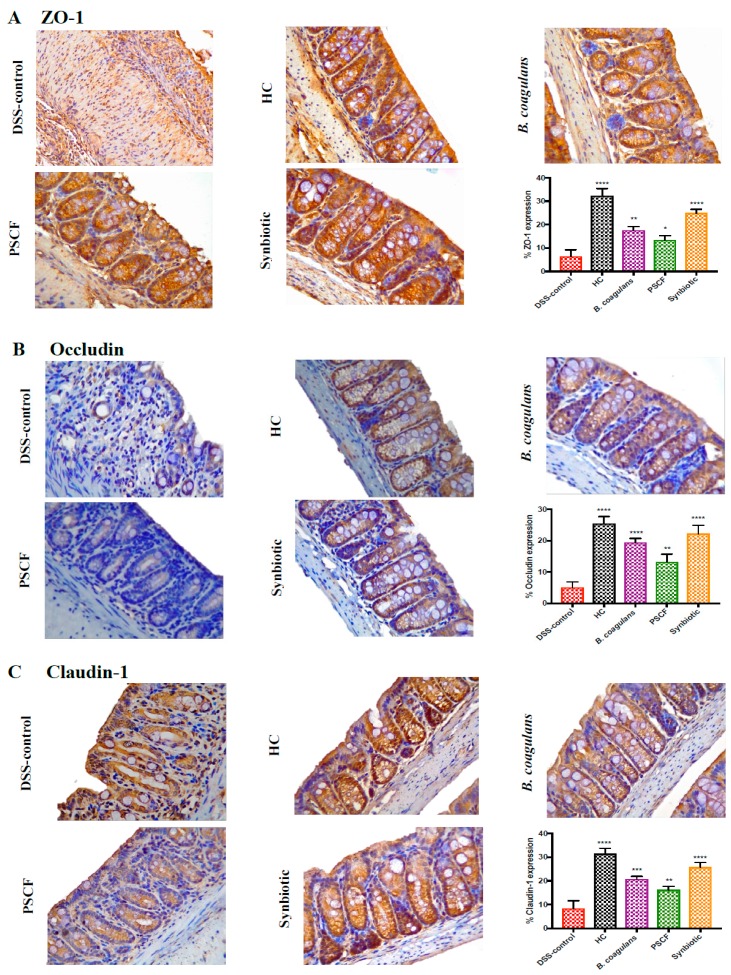
Effects of *B. coagulans* spores, PSCF and synbiotic on expression of epithelial tight junction proteins. Immunohistochemical detection of (**A**) ZO-1, (**B**) Occludin and (**C**) claudin-1 and its respective percentage of expression in colon at 40×. Data expressed as mean ± SEM (*n* = 4 per group) and statistical significance among groups evaluated by one-way ANOVA followed by Tukey’s test *p < 0.05, **p < 0.01, ***p < 0.001, ****p < 0.0001 vs. DSS-control group.

Immunohistochemical analysis was then performed to evaluate the assembly of the TJs and the integrity of the intestinal barrier. The presence of the TJ proteins-ZO-1, occludin, and claudin-1 on the tissue sections were analysed (Figure 4). In HC, ZO-1 (Figure 4A) staining was more intense in the apical tight junction complex both at the surface and in the crypts. In addition to showing their presence at the crypt surface, occludin (Figure 4B), and claudin-1 (Figure 4C) proteins stained more strongly at the basolateral membrane of crypts. As previously reported [38,39] such signals were weak or totally absent on the epithelium of DSS-control sections resulting in very low percentage TJ protein expressions. Basolateral and partial apical staining of ZO-1, occludin and claudin-1 was maintained with *B. coagulans* and synbiotic supplementation in DSS-treated animals. While PSCF was able to partially maintain ZO-1 and claudin-1 staining, such an effect was less evident for occludin. Synbiotic treatment was most effective in preserving the TJ protein expressions in DSS-induced mice further confirming its beneficial effects on the intestinal integrity.

### 3.4. Immunomodulatory Effects of B. Coagulans, PSCF, and Synbiotic Supplementation on Immune Markers in DSS-Induced Mice

**Figure 5:**
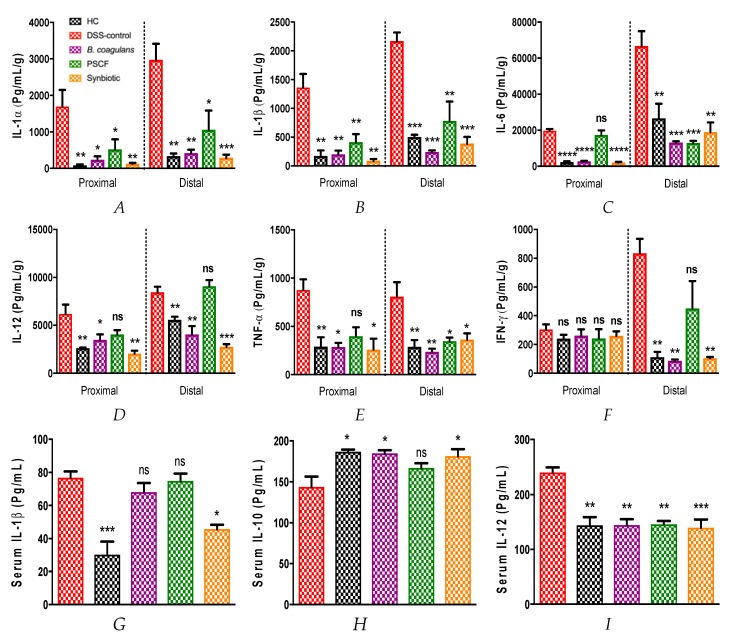

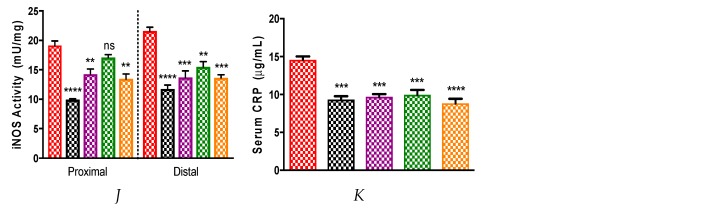
Effect of *B. coagulans* spores, PSCF and synbiotic on immune markers in colon tissues and blood serum. Protein levels of cytokines including (**A**) IL-1α, (**B**) IL-1β, (**C**) IL-6, (**D**) IL-12, (**E**) TNF-α, (**F**) IFN-γ in proximal and distal colon explants as well as cytokine levels of (**G**) IL-1β, (**H**) IL-10, and (**I**) IL-12 in blood serum were analysed by Bio-plex. iNOS activity in colon tissues (**J**) measured by NOS activity assay and CRP levels in serum (**K**) by ELISA. Statistical significance among groups evaluated by one-way ANOVA followed by Tukey’s test. *p < 0.05, **p < 0.01, ***p < 0.001, ****p < 0.0001 vs. DSS-colitic group and data expressed as mean ± SEM (*n* = 3 per group).

The cytokine analysis of the colonic segments and serum measured the intestinal immunomodulatory and anti-inflammatory effects of *B. coagulans*, PSCF and their synbiotic combination. A beneficial impact of ameliorating the altered immune response induced by DSS intake was noted. Overall, probiotic, prebiotic, and synbiotic treatments remarkably reduced the pro-inflammatory cytokine secretions of IL-1α, IL-1β, IL-6, IL-12, TNF-α, and IFN-γ in proximal and distal colon segments compared with that of the DSS-colitic segments (Figure 5), while no significant effect of supplementations was noted on other cytokine levels (Supplementary Figure S1). *B. coagulans* and synbiotic treatments were equally effective in maintaining the levels of these altered cytokines relative to that of non-colitic mice while PSCF did not show significant effective reduction of IL-12 cytokine levels (Figure 5D). Synbiotic treatment was statistically more potent in suppressing the elevated levels of IL-1α (−90.29%) and IL-12 (−67.42%) compared with *B. coagulans* (−85.94%, −52.20%, respectively) in the DC. No positive effect was observed in the PC segment for IL-6 and TNF-α levels by PSCF treatment. However, the excellent immunomodulatory effect in the DC for respective cytokines was confirmed (Figure 5C,E), implicating its differential effects in these colonic segments. 

Similar immunomodulatory effects were found on the serum cytokines Il-1β, IL-12, and IL-10 (Figure 5G–I), while no significant effect was observed for other serum cytokines (Supplementary Figure S1). All three treatments substantially restored the IL-12 levels to values similar to HC mice, while synbiotic treatment was significantly more efficacious in reducing pro-inflammatory IL-1β levels in serum. Moreover, *B. coagulans* and synbiotic treatments increased the anti-inflammatory IL-10 levels in serum (181.2 ± 8.70, 184.7 ± 3.81 pg/mL respectively) compared with the DSS-colitic group (143.8 ± 12.80 pg/mL) (Figure 3H). iNOS activity which is known to be high in colitis in response to pro-inflammatory stimuli [33], was significantly suppressed in both colon segments by all treatments (Figure 5J) compared with DSS-colitic levels. Serum CRP level was significantly higher in the DSS-control group (14.58 ± 0.45 μg/mL) in comparison with HC animals (9.32 ± 0.45 μg/mL). Bacillus, PSCF and synbiotic remarkably normalised the CRP levels (9.67 ± 0.34, 9.95 ± 0.65, 8.83 ± 0.59 μg/mL, respectively) to that of the HC group. These results confirmed the notable anti-inflammatory efficacy of probiotic *Bacillus* spores, prebiotic PSCF and synbiotic treatments used in our study. 

### 3.5. Effects of B. coagulans, PSCF and Synbiotic Supplementation on Altered Fecal Metabolic Profile in DSS-Induced Mice

**Figure 6:**
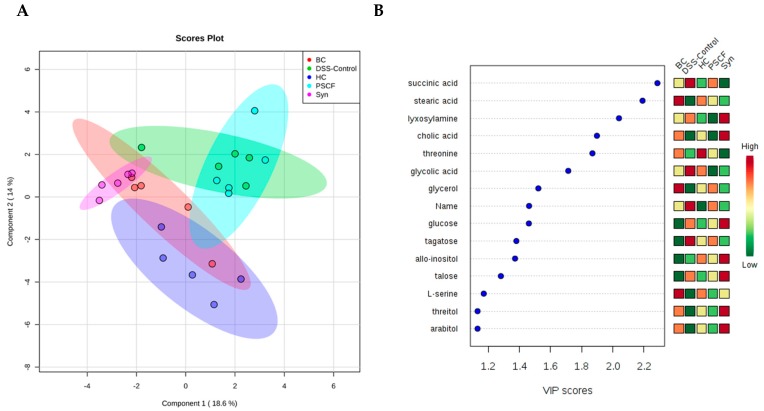
Effect of *B. coagulans* spores, PSCF and synbiotic on metabolic modulations in DSS-induced colitic mice. (**A**) 2D-PLS-DA plot showing spatial division among groups that received different supplementations, DSS-control mice that received no supplementation and HC. (**B**) Key compounds separating the groups based on variable importance projection (VIP) score plot in PLS-DA analysis. (BC-*B. coagulans*, Syn-synbiotic).

Fecal metabolites were analysed using GC-MS to gain an overview of changes induced by *B. coagulans*, PSCF and synbiotic supplementation in DSS-treated mice. A total of 61 metabolites of different functional groups such as sugars, amino acids, volatile fatty acids, and biogenic amines were detected. A supervised partial least squares-discriminant analysis (PLS-DA) was performed to evaluate metabolic phenotyping of each experimental group as shown in Figure 6A. The samples from HC and DSS-control clusters were clearly divergent indicating marked distinction in metabolic patterns between the two groups. While, clusters of supplemented mice samples overlapped with that of HC and DSS-control, clear demarcation of the synbiotic and *B. coagulans* cluster was noted, with PSCF showing only partial divergence relative to that of DSS-control. This indicates substantial efficacy of *B. coagulans* to metabolise PSCF that, in turn, induced significant biochemical changes potentially owing to their synergistic effects as evidenced from synbiotic samples. Combination of PLS-DA (R^2^Y = 0.810 (*p* = 0.01), Q^2^ = 0.710), VIP scores (Figure 6B) and significance analysis for microarrays (SAM) enabled us to identify potential biomarkers. The results showed 61 metabolites with 40 statistically significant compounds contributing to the clustering, with their SAM scores fold changes and International Chemical Identifiers (InChI) and standard InChI hashes (InChIKey IDs) listed in Table S1. Key metabolic markers making a significant contribution were identified by VIP analysis as displayed in Figure 6B. Among these identified metabolites, noticeable differences between DSS-control, and HC samples were noted particularly for succinic acid, stearic acid, and glycerol. Synbiotic supplementation was beneficial in minimising the metabolite alterations induced by DSS (Figure 6B and Table S1). 

### 3.6. Effects of B. Coagulans, PSCF and Synbiotic Supplementation SCFA Levels in DSS-Induced Mice

**Figure 7:**
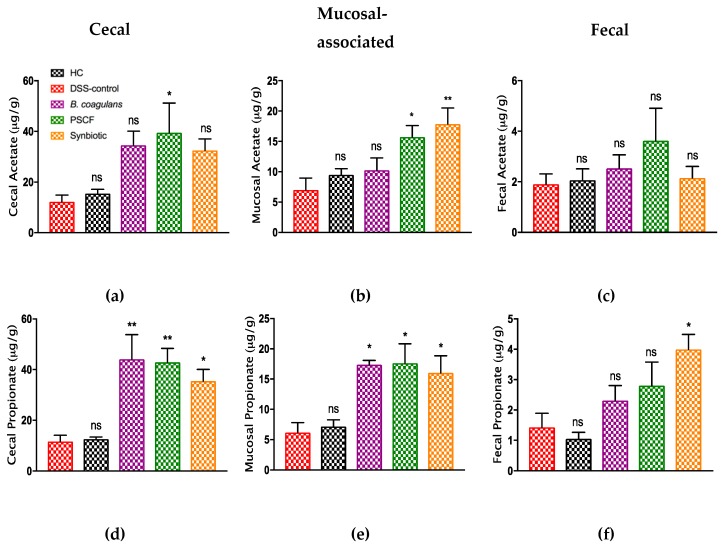

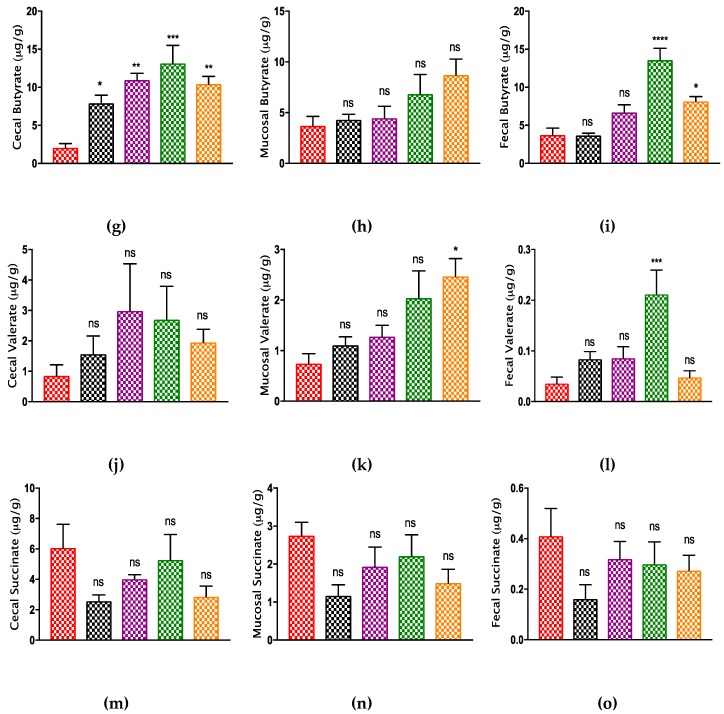
Effects of *B. coagulans* spores, PSCF and synbiotic in modulating SCFA concentrations in cecal, mucosal-associated and fecal contents in DSS-induced colitis. Cecal- acetate (**a**), proionate (**d**), butyrate (**g**), valerate (**j**), succinate (**m**); mucosal-associated acetate (**b**), proionate (**e**), butyrate (**h**), valerate (**k**), succinate (**n**) and fecal- acetate (**c**), propionate (**f**), butyrate (**i**), valerate (**l**), succinate (**o**). -Statistical significance among groups evaluated by one-way ANOVA followed by Tukey’s test. *p < 0.05, **p < 0.01, ***p < 0.001, ****p < 0.0001 vs. DSS-colitic group and data expressed as mean ± SEM (*n* = 5 per group). ns-non-significant

As shown in Figure 7, supplementation of DSS-induced mice with *B. coagulans*, PSCF, and synbiotic treatments induced substantial modulations in the SCFA concentrations and their effects varied across cecal, luminal and fecal contents. Overall, the highest concentration of SCFA were noted in cecal contents compared to mucosal and fecal contents. There were no significant differences between the concentration of acetate and propionate in DSS-control and HC mice. PSCF however, induced a significant increase in the acetate levels in cecal and mucosal samples while, Synbiotic treatment was most significant in elevating acetate concentrations in mucosal-associated samples. All three supplementations were significantly effective in improving the propionate concentrations in cecal and mucosal contents while, only synbiotic supplementation increased propionate levels in fecal samples.

Butyrate levels were significantly decreased by DSS administration (2.05 ± 0.6 μg/g) compared with that of HC mice (7.91 ± 1.1 μg/g) in the cecum. The decrease in butyrate induced by DSS was maintained at control levels with all three supplements in the cecum with PSCF (13.2 ± 2.4 μg/g) being significantly more effective than *B. coagulans* (11 ± 0.9 μg/g) and synbiotic (10.4 ± 1.0 μg/g). Similarly, in fecal content, PSCF significantly improved butyrate (13.6 ± 1.6 μg/g compared with 3.72 ± 0.1 μg/g for DSS-colitic followed by synbiotic (8.15 ± 0.6 μg/g) while *B. coagulans* (6.69 ± 0.1 μg/g) had no effect. Synbiotic supplementation resulted in marked increase in mucosal-assocaited valerate while, PSCF caused elevation in its concentration in fecal content. Similarly, synbiotic was more efficient in reducing elevated succinate levels. 

## 4. Discussion

Dietary strategies involving probiotic and prebiotic fibre components that function by modulating immune responses, colonic epithelial integrity and microbial composition and related metabolites are being widely investigated for the prevention or reduction of severity of IBD [40,41]. The present study clearly supported the premise that conditioning of the gut with synbiotic supplementation containing compatible probiotic and prebiotic fibre can be greatly beneficial to reducing the symptoms and severity of DSS-induced acute colitis in mice. The observations confirmed substantial anti-inflammatory efficacy by synbiotic supplementation containing *B. coagulans* and PSCF. This was evidenced by the improvement of clinical symptoms, macroscopic, histological, biochemical, metabolic and immune parameters in the DSS-induced colitic mice model.

The addition of 2% (*w*/*v*) DSS to drinking water for seven days without ameliorating treatments resulted in a progressive rise in DAI (Figure 1A), owing to both body weight loss (Figure 1B) and excretion of diarrheic/bleeding feces. However, the supplementation of DSS-induced mice with *B. coagulans*, PSCF, and their synbiotic combination significantly attenuated the severity of the DSS damage and improved DAI and macroscopic markers of inflammation (Figure 1C–F). The ability of PSCF to show early effect on DAI and body weight could be related to its high content of insoluble hemicellulose. This fraction has a large water-holding capacity thus, could appropriately contribute to regulating the fecal water content in colitic mice [42,43]. The anti-diarrheal effect of *B. coagulans* have been previously confirmed [44]. The increased beneficial effects of synbiotic supplementation in reducing the disease severity could be related to the synergistic actions between the probiotic and prebiotic components.

A potentiated synbiotic effect relative to that of *B. coagulans* and PSCF individually was also evident from the histology of the colon compared with DSS-control mice (Figure 2A,B). Synbiotic supplementation showed substantial protection to the colonic epithelial architecture by mitigating crypt disruption, loss of goblet cells, submucosal edema and epithelial structure damage induced by DSS. Synbiotic supplementation also induced suppression of infiltration of activated neutrophils as evidenced by significant reduction in MPO activity in DSS-induced mice (Figure 2C). The infiltration of activated neutrophils is one of the most prominent histological features observed in IBD and is directly proportional to the MPO activity. Superoxide anions and other reactive species produced by neutrophils leads to tissue necrosis and mucosal dysfunction in IBD [39]. The reduction in MPO activity suggests that synbiotic supplementation imparted an anti-inflammatory effect, in addition to histological protection.

Disruption of intestinal epithelial TJs and impaired epithelial barrier function is a central event in the pathogenesis of IBD and may lead to persistent aberrant immune reactions, thus accelerating gut inflammation and the inflammatory circuit [45]. Synbiotic treatment in our study was the most effective followed by *B. coagulans* and PSCF in protecting the expression level of TJ proteins (ZO-1, occludin and claudin-1) in DSS-induced mice (Figure 4). TJs create a semi-permeable barrier against paracellular penetration of harmful substances from the gut lumen [45]. *Bacillus subtilis* intake has been recently confirmed to upregulate the expressions of TJ proteins for improved barrier function in colitic mice [46] and corroborates with our results. Moreover, synbiotic and *B. coagulans* protected the goblet cells and mucin production more effectively than PSCF alone (Figure 3). This indicates the possible ability of these *B. coagulans* MTCC 5856 spores to benefit goblet cell structure and function and needs further investigation to determine the mechanism of this effect. Some *Bacillus* species have been shown to upregulate mucin glycoproteins and protect colonic mucus layer integrity and goblet cell function [47]. Although we could not confirm if synbiotic supplementation had a stimulating effect on TJ proteins and/or localisation or, instead if it avoided TJ degradation by DSS, we observed that synbiotic supplementation significantly maintained the TJ patterns similar to that of animals in HC. This is indicative that synbiotic supplementation significantly preserved the integrity of the epithelium. The synergy between *B. coagulans* and PSCF could have imparted excellent protection and/or maintenance of epithelial integrity on DSS-induced mice, thus supporting its application in IBD to reinforce intestinal barrier integrity. 

Alterations in the barrier integrity in IBD also leads to aberrant immune responses resulting in an inflammation cascade and tissue damage [48]. Although *B. coagulans* and PSCF supplementations alone were able to modulate the tested cytokines (IL-1α, IL-1β, IL-6, IL-12, TNF-α, and IFN-γ), a more profound anti-inflammatory effect was observed with synbiotic supplementation in both the colon and serum (Figure 5A–I). A spike in the levels of IL-1β, IL-6, and TNF-α have been implicated in human IBD pathogenesis [49]. Such pro-inflammatory cytokines are secreted at high levels by activated lamina propria antigen presenting cells in response to inflammation. Pro-inflammatory cytokines such as IL-6, TNF-α, and IL-1β are being targeted by therapeutic approaches to curb the aberrant inflammatory response in IBD due to their roles in the pathogenesis of the disease [50,51,52]. TNF-α is reported to exert its pro-inflammatory effect through elevated production of IL-6 and IL-1β [53]. This is in line with the present study, where DSS-induction caused elevated secretions of these cytokines in DSS-control group relative to that of HC (Figure 5). Upregulation of pro-inflammatory cytokine levels has also been reported to upregulate iNOS expression and secretion of nitric oxide that causes tissue damage in IBD [54]. The anti-inflammatory effects of synbiotic, *B. coagulans* and PSCF in suppressing the levels of these pro-inflammatory cytokines as well as reducing iNOS activity in colonic tissues, indicates their immunomodulatory potentials. *B. coagulans* MTCC 5856 spores in our previous study showed excellent immunomodulatory effect in vitro [22]. Marked reduction in pro-inflammatory cytokines in the colon of DSS-induced mice in the current study further supports its excellent immunomodulatory efficacy in IBD application. 

Synbiotic supplementation was the most effective in demonstrating noticeable anti-inflammatory effect by significantly reducing the serum levels of pro-inflammatory IL-1β and IL-12 while concurrently elevating anti-inflammatory IL-10 in the serum. IL-10 plays a prominent role in counterbalancing Th1 and Th17 immune activity in IBD towards a Th2 response by downregulating antigen presentation and subsequent release of proinflammatory cytokines thereby attenuating mucosal inflammation [53]. IL-10 has been reported to play a role in maintaining intestinal barrier integrity possibly owing to effects on zonulin pathway [55]. The ability of *B. coagulans* spores to elicit IL-10 levels in inflammatory condition has been determined in vitro and in human subjects. Thus, the anti-inflammatory efficacy of synbiotic supplementation could be related to major immune-regulating capability of *B. coagulans* MTCC 5856 spores, thereby supporting its application in synbiotic therapies for IBD. Furthermore, synbiotic treatment also reduced the increased CRP levels in the serum of DSS-induced mice. In the inflammatory state, circulating IL-6 promotes CRP production in the liver and its release into the bloodstream [56]. Elevated levels of CRP has been implicated in human IBD patients [57]. The reduction in overall pro-inflammatory mediators by synbiotic supplementation may be due to either direct immune-regulating effect of the *B. coagulans* and PSCF on cytokine secretion or it could be owing to their indirect effect on the protection of intestinal barrier integrity. In either case this leads to reduction in luminal antigens and full activation of the innate immune system. 

DNA extracted from cecal, fecal and mucosal-associated contents from DSS-induced mice in the present study, failed amplification before 16s rRNA sequencing owing to the presence of DSS in the samples that inhibited the PCR amplification as known earlier [58]. The microbiota profiling of DSS samples could therefore not be performed and is the limitation of the study. The levels of microbiota-derived untargeted metabolites and SCFAs were therefore alternatively analysed as signatures of the gut microbiota that contribute to modulating the immune activity of the intestinal mucosa [59,60]. The untreated DSS-control mice exhibited distinct fecal metabolic phenotype relative to that of HC. This was reflective of clinical and animal IBD studies that confirmed significant differences in metabolic profiles between healthy and IBD patients [61,62,63]. The metabolic analysis of supplemented mice in our study resulted in considerable normalization of metabolic profile indicating the positive effects of synergistic combination of *B. coagulans* and PSCF that induced marked improvement in metabolic pattern. Notably for mice supplemented with PSCF there was not much distinction from the DSS-control group was observed but the synbiotic combination with *B. coagulans* resulted in an improved metabolic pattern. This marked synergism could be associated with the acceleration of fermentation of insoluble plant cellular materials, such as hemicellulose in PSCF, by the supplemented *B. coagulans* [18]. The resulting higher levels of fermentation metabolites would thus, in turn, influence other beneficial microbial metabolic activities. This further supports the application of compatible synbiotic components to generate maximum benefits through increased SCFA production. 

Increased levels of microbiota-derived SCFAs are inversely associated with dysbiosis in IBD [64]. Of particular interest are higher levels of acetate, propionate and butyrate, which results from fermentation of indigestible carbohydrates from fibre-rich diets. The effects of SCFAs have been studied to in animal models of colitis [42] and clinical UC [65]. Each type of SCFA is likely to contribute to host health [66]. In this study we determined the SCFA profile along the gastro-intestinal tract, analyzing samples across cecal, mucosal-associated, and fecal contents. The concentration of SCFAs varied along the length of the gut, with most abundant levels in the cecum and PC, while it declined towards the DC [66]. In line with a recent study [67], cecum showed the highest levels of all the SCFAs tested in our study irrespective of the supplementation. Cecum is considered the major site for fermentation in the rodent gut and contains the largest pool of microbiota. It therefore generates the most SCFAs. However, some recent studies have also reported changes in the microbiota and associated amounts of metabolites in along different regions of gastro-intestinal (GI) tract [67,68,69]. Therefore, the overall GI profile is of importance when associating the gut microbiome and metabolites with health outcomes. 

All three supplementations caused a substantial increase in concentrations of measured SCFAs compares with that of DSS-control group. However, the potentiated effect of synbiotic supplementation is evidenced by induction of SCFA production along the entire length of the colon compared to *B. coagulans* supplementation alone where the additional SCFA production capacity was absent past the cecum. This effect highlights the advantage of using a compatible prebiotic fibre as synbiotic companion with a particular probiotic that can metabolize it. The high total dietary fibre content of PSCF (87%) [17] would contribute to this effect. PSCF supplementation resulted in increased butyrate levels line with an in-vitro study with human gut microbiota utilizing sugar cane fibre [17]. The effect of additional SCFA production in cecum by *B. coagulans* would not extend to the proximal or distal parts of the colon. In the current study, the elicited extra SCFAs with synbiotic supplementation from cecum to the fecal pellets indicated the ability of *B. coagulans* to utilize the PSCF to also generate SCFAs after the cecum. The ability of *B. coagulans* to metabolize a variety of plant fibers for fermentation, including cranberry fibre [70] and fenugreek seeds [71], to produce SCFAs and hemicellulose [18] for lactic acid production has been previously determined. 

PSCF supplementation also resulted in increased butyrate levels, correlating with results of an in-vitro study with human gut microbiota utilizing sugar cane fibre [17]. This ability of synbiotic supplementation for eliciting butyrate levels along the entire length of colon could contribute to the beneficial effect observed in the current study. Butyrate is the preferred energy source for colonocytes and has the ability to regulate cytokines thus showing protection against inflammation in UC and colorectal cancer [66]. Butyrate has been demonstrated in in vitro [72,73] and in vivo [74] studies to increase epithelial integrity and mucus secretion, consistent with the immunohistological and mucus staining analysis in the present study. The substantial increase in butyrate levels in the cecum by *B. coagulans* supplementation may be due to ability to support the growth and activity of butyrate producers probably via cross-feeding of the lactic acid production. *B. coagulans* are known to be efficient at producing lactic acid through fermentation of various plant substrates, including hemicellulose [18,19]. Lactic acid is reported to be utilized by strictly anaerobic butyrate-producing bacteria of clostridial clusters XIVa for the production of high concentrations of butyric acid [75]. Thus, the synbiotic approach with probiotic bacteria and prebiotic fibre that directly or indirectly influence butyrate production may help to restore intestinal barrier integrity in diseased state. This effect was evidenced by the significant reduction in DSS-induced colonic epithelial damage (Figure 2) by synbiotic supplementation in our study.

Acetate and propionate have also been studied to benefit epithelial integrity via binding with certain metabolite-sensing G-protein-coupled receptors (such as GPR43, GPR109A) and modulating immune response [76,77,78]. Valerate that has been determined to stimulate intestinal growth and attenuate inflammatory pathogenesis in colitis and cancer to metabolic disorders [79] was increased by synbiotic supplementation. Besides its positive effect in colon, SCFAs have also been exhibited to mediate improved host metabolism and modulate the activity of the enteric nervous system [66], thus providing benefits beyond GI tract. The high levels of immuno-modulatory effects observed in the present study could also possibly be correlated to high SCFA levels induced by synbiotic supplementation owing to the synergistic combination. SCFAs by engaging with engage with GPRs are known to induce immune-modulation leading to a direct local and systemic anti-inflammatory effects [76,80]. This further supports the application of synergistic synbiotic combinations to achieve maximum benefits in resolving the inflammatory circuit in IBD.

## 5. Conclusions

This is a detailed study highlighting the site-specific inflammatory and SCFA changes in a mice model of IBD as a result of synbiotic supplementation of the normal diet with prebiotic whole plant fibre and probiotic spores. The synbiotic pre-supplementation resulted in a substantial anti-inflammatory effect, reducing disease severity, colonic damage, and inflammatory mediators while modulating the metabolite and SCFA profiles of DSS-induced gut damage. The research has clearly demonstrated that the supplementation of whole plant PSCF and *B. coagulans* spores produced a synergistic combination that protected mice against acute damage induced by DSS in mice. The results underscore the significant efficacy of synbiotic applications to increase the beneficial and preventive effects on the host by targeting different mechanistic approaches to resolve inflammation cycle. However, the differences in the evolved biology of humans compared to mice requires caution in translation of the results to impacts on human disease [81]. While mice models do allow the changes in gut microbiota, as a result of pre- and probiotic combinations to be studied in a controlled experiment direct human trials will be needed. The delineation of the synergistic biological actions of probiotic *B. coagulans* spores and prebiotic PSCF in mouse model of IBD provides support for investigating their therapeutic and preventive effects in human IBD. However, the ability to reduce the severity of DSS-induced colitis was demonstrated using pre-supplementation. Human trials should be aimed at proactive prevention or after partial control of inflammatory disease, such as in association with drug treatment. 

Patents: The information from this study has been filed as a provisional patent application in Australia titled “Preparation for the Treatment of Inflammatory Bowel Disease using a Whole Plant Fibre Extract from Sugar Cane” with application number 2018902145 and a filing date of 15 June 2018. Information relating to novelty of synergy between probiotic *Bacillus coagulans* and prebiotic whole plant sugar cane fibre in imparting health benefits is the subject of the patented claim.

## Supplementary Materials

The following are available online at https://www.mdpi.com/2072-6643/11/4/818/s1, Figure S1: Effect of B. coagulans spores, PSCF and synbiotic on immune markers in colon tissues and blood serum, Table S1: Most significant compounds identified by OPLS-DA and SAM analysis in HC, DSS-control, B. coagulans (BC), PSCF and synbiotic groups.
