# Supplementary material for: Synbiotic Supplementation Containing Whole Plant Sugar Cane Fibre and Probiotic Spores Potentiates Protective Synergistic Effects in Mouse Model of IBD
Source: Nutrients. 2019 Apr 11;11(4):818. doi: 10.3390/nu11040818 (PMC6521199; doi:10.3390/nu11040818)
Supplement: Supplementary file 1 [file nutrients-11-00818-s001.pdf]

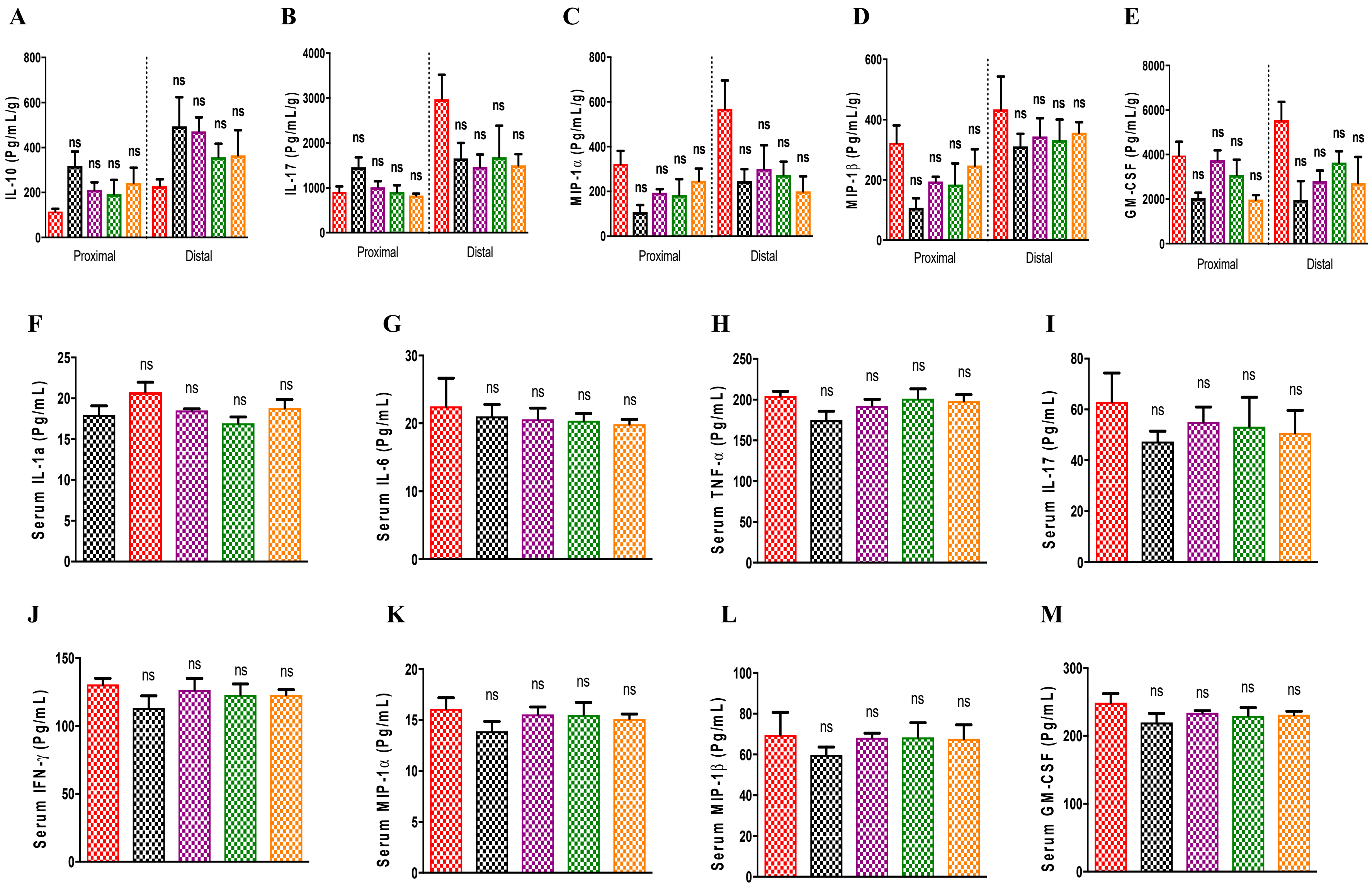
A
IL-10 (Pg/mL/g)
B
IL-17 (Pg/mL/g)
C
MIP-1α (Pg/mL/g)
D
MIP-1β (Pg/mL/g)
E
GM-CSF (Pg/mL/g)
Proximal
Distal
ns
F
Serum IL-1a (Pg/mL)
G
Serum IL-6 (Pg/mL)
H
Serum TNF-α (Pg/mL)
I
Serum IL-17 (Pg/mL)
J
Serum IFN-γ (Pg/mL)
K
Serum MIP-1α (Pg/mL)
L
Serum MIP-1β (Pg/mL)
M
Serum GM-CSF (Pg/mL)

Figure S1. Effect of *B. coagulans* spores, PSCF and synbiotic on immune markers in colon tissues and blood serum. Protein levels of cytokines including (A) IL-10 (B) IL-17, (C) MIP-1α, (D) MIP-1β, (E) GM-CSF in proximal and distal colon explants as well as cytokine levels of (F) IL-1α, (G) IL-6, (H) TNF-α, (I) IL-17, (J) IFN-γ, (K) MIP-1α, (L) MIP-1β, (M) GM-CSF in blood serum were analysed by Bio-plex. Statistical significance among groups evaluated by one-way ANOVA followed by Tukey's test. Non-significant (ns) vs. DSS-colitic group and data expressed as mean ± SEM (n = 3 per group).

Table S1. Most significant compounds identified by OPLS-DA and SAM analysis in HC, DSS-control, *B. coagulans* (BC), PSCF and synbiotic groups (*First 40 compounds are identified by SAM).

Compound name	InCHI Key	DSS-control (FC)	BC (FC)	HC (FC)	PSCF (FC)	Synbiotic (FC)	SAM (p value)
Oxalic acid*	KZSNJWFQEVHDMF-BYPYZUCNSA-N	1.3174	1.255	0.55868	1.1763	1.1165	0.00098361
Urea*	XSQUKJJJFZCRTK-UHFFFAOYSA-N	1.8432	1.6662	1.0792	1.6115	1.5227	0.00098361
Allantoin*	POJWUDADGALRAB-UHFFFAOYSA-N	2.148	1.9001	0.68545	1.7891	1.704	0.0029508
Stearic acid*	QIQXTHQIDYTFRH-UHFFFAOYSA-N	1.8728	1.7465	2.1912	1.6452	1.5837	0.0031148
Lyxosylamine*	RQBSUMJKSOSGJJ-AGQMPKSLSA-N	1.582	1.4033	2.0398	1.3617	1.3011	0.0057377
L-proline*	ONIBWKKTOPOVIA-BYPYZUCNSA-N	0.15132	0.33928	0.11327	0.33732	0.53724	0.0090164
Threonine*	AYFVYJQAPQTCCC-GBXIJSLDSA-N	1.4467	1.2792	1.8691	1.242	1.2061	0.0096721
L-alanine*	MUBZPKHOEPUJKR-UHFFFAOYSA-N	1.0679	1.0122	0.8546	1.0102	0.96143	0.011803
Uracil*	ISAKRJDGNUQOIC-UHFFFAOYSA-N	0.17897	0.366	0.22317	0.34487	0.70605	0.015902
Cholic acid*	BHQCQFFYRZLCQQ-OELDTZBJSA-N	1.5243	1.3972	1.8978	1.3157	1.2664	0.020328
Glycerol*	PEDCQBHIVMGVHV-UHFFFAOYSA-N	1.1775	1.1305	1.5212	1.0639	1.0054	0.021148
Myristic acid*	TUNFSRHWOTWDNC-UHFFFAOYSA-N	0.054605	0.46469	0.070724	0.5799	0.56785	0.023279
Cholesterol*	HVYWMOMLDIMFJA-DPAQBDIFSA-N	0.52261	0.77314	0.17624	0.72764	0.70976	0.027377
Tagatose*	LKDRXBCSQODPBY-OEXCPVAWSA-N	1.1157	1.245	1.38	1.1698	1.113	0.028852
Phenylethylamine*	BHHGXPLMPWCGHP-UHFFFAOYSA-N	1.0647	1.5462	1.0064	1.4544	1.5756	0.032787
Hypoxanthine*	FDGQSTZJBFJUBT-UHFFFAOYSA-N	0.096521	0.28394	0.11887	0.26659	0.58207	0.032951
Palmitic acid*	IPCSVZSSVZVIGE-UHFFFAOYSA-N	0.49285	0.74893	0.58984	0.73808	0.70058	0.037049

L-norleucine*	LRQKBLKVPFOOQJ-YFKPBYRVSA-N	0.8472	0.77466	0.93985	0.79056	0.76421	0.039508
L-valine*	KZSNJWFQEVHDMF-BYPYZUCNSA-N	0.77076	0.70997	0.99092	0.67789	0.72327	0.042951
Sorbose*	LKDRXBCSQODPBY-AMVSKUEXSA-N	0.6984	1.0726	0.90204	1.0223	0.96654	0.044754
Succinic acid*	KDYFGRWQOYBRFD-UHFFFAOYSA-N	2.0091	1.9716	2.2865	1.8752	1.7868	0.044918
Linoleic acid*	OYHQOLUKZRVURQ-HZJYTTRNSA-N	0.98508	0.91011	0.82359	0.95291	0.90628	0.04623
Iminodiacetic acid*	NBZBKCUXIYYUSX-UHFFFAOYSA-N	0.34183	0.51782	0.39691	0.51168	0.49391	0.053115
Glycine*	DHMQDGOQFOQNFH-UHFFFAOYSA-N	0.83115	0.76955	0.25675	0.73528	0.7	0.063115
Arabitol*	HEBKCHPVOIAQTA-QWWZWVQMSA-N	1.2402	1.3004	1.1307	1.2202	1.1702	0.071967
Threitol*	UNXHWFMMPAWVPI-QWWZWVQMSA-N	1.2402	1.3004	1.1307	1.2202	1.1702	0.071967
Name*	-	1.4617	1.2793	1.4356	1.261	1.2628	0.084426
Cellobiose2*	DLRVVLDZNNYCBX-ABXHMFFYSA-N	1.0521	1.1194	0.36794	1.177	1.2056	0.087705
Cellobiose1*	DLRVVLDZNNYCBX-ABXHMFFYSA-N	1.0965	1.1616	0.27576	1.2031	1.2329	0.08918
L-lactic acid*	JVTAAEKCZFNVCJ-REOHCLBHSA-N	0.38064	0.34109	0.42171	0.34038	0.54515	0.098361
Nicotinic acid*	PVNIIMVLHYAWGP-UHFFFAOYSA-N	0.2932	0.42574	0.043319	0.44966	0.80456	0.10361
Glucose*	WQZGKKKJIJFFOK-GASJEMHNSA-N	1.1379	1.0103	1.4604	0.95824	1.0583	0.10869
Talose*	WQZGKKKJIJFFOK-WHZQZERISA-N	0.98914	0.89045	1.2805	0.8523	0.97672	0.11852
Phosphoric acid*	NBIIXXVUZAFLBC-UHFFFAOYSA-N	0.94288	0.89642	1.0701	0.89948	0.85042	0.13213
Oleic acid*	ZQPPMHVWECSIRJ-KTKRTIGZSA-N	0.82774	0.8867	0.87563	0.8825	0.84222	0.13557
Allose*	WQZGKKKJIJFFOK-IVMDWMLBSA-N	0.73394	0.97555	0.93891	0.99147	0.93876	0.13656
Altrose*	WQZGKKKJIJFFOK-VSOAQEOCSA-N	0.50595	0.92205	0.39772	1.089	1.0305	0.13852
Benzoic acid*	WPYMKLBDIGXBTP-UHFFFAOYSA-N	1.0249	0.91542	0.77027	0.93124	0.89526	0.14951
Lactose*	GUBGYTABKSRVRQ-DCSYEGIMSA-N	0.87631	0.91851	0.98755	1.1343	1.1067	0.15033
Melibiose*	DLRVVLDZNNYCBX-ABXHMFFYSA-N	0.88773	0.92323	1.0214	1.1454	1.1147	0.15918
Glycolic acid	AEMRFAOFKBGASW-UHFFFAOYSA-N	1.7136	1.7406	1.9566	1.6458	1.5606	
DL-isoleucine	AGPKZVBTJJNPAG-UHFFFAOYSA-N	0.26715	0.26613	0.33241	0.43773	0.41604	
Glyceric acid	RBNPOMFGQQGHHO-UWTATZPHSA-N	0.23877	0.2287	0.17023	0.23149	0.52183	
L-serine	MTCFGRXMJLQNBG-REOHCLBHSA-N	0.90573	0.80824	1.1704	0.76565	0.72921	

Thymine	RWQNBRDOKXIBIV-UHFFFAOYSA-N	0.2904	0.60831	0.13184	0.66579	0.85964	
Malonic acid	OFOBLEOULBTSOW-UHFFFAOYSA-N	0.91188	0.81386	0.88259	0.77588	0.74087	
Methionine	FFEARJCKVFRZRR-BYPYZUCNSA-N	1.0606	1.0069	1.0096	0.9487	0.90453	
Aspartic acid	CKLJMWTZIZZHCS-REOHCLBHSA-N	0.47085	0.41676	0.57776	0.73065	0.74922	
4-guanidinobutyric acid	TUHVEAJXIMEOSA-UHFFFAOYSA-N	0.23097	0.3392	0.13804	0.32329	0.32754	
Alpha ketoglutaric acid	KPGXRSRHYNQIFN-UHFFFAOYSA-N	0.70868	0.79797	0.85838	1.0383	0.98219	
Glutamic acid	WHUUTDBJXJRKMK-VKHMYHEASA-N	0.82114	0.7985	1.0603	0.9751	1.1655	
5-aminovaleric acid	JJMDCOVWQOJGCB-UHFFFAOYSA-N	0.41256	0.68247	0.15453	0.74946	1.0804	
Lyxose	SRBFZHDQGSBBOR-AGQMPKSLSA-N	0.079671	0.071301	0.080884	0.37509	0.61504	
6-deoxy-D-glucose	SHZGCJCMOBCMKK-GASJEMHNSA-N	0.77237	0.79383	0.65727	0.79326	0.7504	
xylitol	HEBKCHPVOIAQTA-NGQZWQHPSA-N	0.5054	0.63101	0.41881	0.63275	0.65337	
Galactose	WQZGKKKJIJFFOK-SVZMEOIVSA-N	0.66763	0.96068	0.84803	0.98453	0.9307	
Tyramine	DZGWFCGJZKJUFP-UHFFFAOYSA-N	0.69223	0.82033	0.59041	0.8402	0.79394	
Lysine	KDXKERNSBIXSRK-YFKPBYRVSA-N	0.42108	0.69937	0.49143	0.65788	0.6221	
Tyrosine	OUYCCCASQSFEME-QMMMGPOBSA-N	0.79626	0.71207	0.74887	0.66747	0.63055	
Allo-inositol	CDAISMWEOUEBRE-UHFFFAOYSA-N	1.194	1.1	1.3715	1.0443	1.0161	
Sucrose	CZMRCDWAGMRECN-UGDNZRGBSA-N	0.58274	0.52774	0.69347	1.0782	1.026	

(International Chemical Identifiers (InChI) and standard InChI hashes (InChIKey); FC = Fold change)
